# Prophylactic Radiologic Interventions for Postpartum Hemorrhage Control in Women With Placenta Accreta Spectrum Disorder: A Systematic Review and Meta-analysis

**DOI:** 10.1097/AOG.0000000000005662

**Published:** 2024-07-02

**Authors:** Lisanne R. Bonsen, Kosma Sleijpen, Joris Hendriks, Thijs A.J. Urlings, Olaf M. Dekkers, Saskia le Cessie, Marc van de Velde, Pema Gurung, Thomas van den Akker, Johanna G. van der Bom, Dacia D.C.A. Henriquez

**Affiliations:** Departments of Obstetrics and Gynaecology, Clinical Epidemiology, Clinical Endocrinology, and Biomedical Data Sciences, Leiden University Medical Center, and Leiden University Libraries, Leiden University, Leiden, the Department of Radiology, Catharina Hospital, Eindhoven, the Department of Radiology, Haaglanden Medical Center, The Hague, and Athena Institute, VU University, Amsterdam, the Netherlands; and the Department of Cardiovascular Sciences, Section Anesthesiology, KU Leuven and UZ Leuven, Leuven, Belgium.

## Abstract

**OBJECTIVE:**

To quantify the association between prophylactic radiologic interventions and perioperative blood loss during cesarean delivery in women with placenta accreta spectrum disorder through a systematic review and network meta-analysis.

**DATA SOURCES:**

On January 3, 2023, a literature search was conducted in PubMed, EMBASE, Cochrane Library, and Web of Science. We also checked ClinicalTrials.gov retrospectively. Prophylactic radiologic interventions to reduce bleeding during cesarean delivery involved preoperative placement of balloon catheters, distal (internal or common iliac arteries) or proximal (abdominal aorta), or sheaths (uterine arteries). The primary outcome was volume of blood loss; secondary outcomes were the number of red blood cell units transfused and adverse events. Studies including women who received an emergency cesarean delivery were excluded.

**METHODS OF STUDY SELECTION:**

Two authors independently screened citations for relevance, extracted data, and assessed the risk of bias of individual studies with the Cochrane Risk of Bias in Non-randomized Studies of Interventions tool.

**TABULTATION, INTEGRATION, AND RESULTS:**

From a total of 1,332 screened studies, 50 were included in the final analysis, comprising 5,962 women. These studies consisted of two randomized controlled trials and 48 observational studies. Thirty studies compared distal balloon occlusion with a control group, with a mean difference in blood loss of −406 mL (95% CI, −645 to −167). Fourteen studies compared proximal balloon occlusion with a control group, with a mean difference of −1,041 mL (95% CI, −1,371 to −710). Sensitivity analysis excluding studies with serious or critical risk of bias provided similar results. Five studies compared uterine artery embolization with a control group, all with serious or critical risk of bias; the mean difference was −936 mL (95% CI, −1,522 to −350). Reported information on adverse events was limited.

**CONCLUSION:**

Although the predominance of observational studies in the included literature warrants caution in interpreting the findings of this meta-analysis, our findings suggest that prophylactic placement of balloon catheters or sheaths before planned cesarean delivery in women with placenta accreta spectrum disorder may, in some cases, substantially reduce perioperative blood loss. Further study is required to quantify the efficacy according to various severities of placenta accreta spectrum disorder and the associated safety of these radiologic interventions.

**SYSTEMATIC REVIEW REGISTRATION:**

PROSPERO, CRD42022320922.

## Abstract 2

Prophylactic placement of balloon catheters or sheaths before planned cesarean delivery in women with placenta accreta spectrum disorder may reduce perioperative blood loss.

Placenta accreta spectrum disorder is a disorder of placentation caused by damage to the endometrial–myometrial interface of the uterus. Placenta accreta spectrum disorder is a high-risk condition in pregnancy, characterized by the failure of placental detachment at the time of birth, potentially leading to life-threatening postpartum hemorrhage.^1,2^ The depth of placental invasiveness is associated with the severity of maternal outcomes, with the severest outcomes in women with placenta percreta.^3,4^

The main risk factor for placenta accreta spectrum disorder is a previous cesarean delivery. Although the incidence of placenta accreta spectrum disorder has increased alongside rising rates of cesarean delivery worldwide, placenta accreta spectrum disorder still remains a rare complication of pregnancy in most settings.^1,5–9^ Therefore, performing robust research to define intrapartum management strategies for women with placenta accreta spectrum disorder to improve maternal outcomes remains challenging.^2,10,11^

One strategy to reduce perioperative bleeding in women at risk of placenta accreta spectrum disorder is the use of prophylactic radiologic interventions, which include preoperative placement of arterial balloon catheters or sheaths by an interventional radiologist. Inflation of the balloons or embolization directly after childbirth is hypothesized to reduce blood flow to the uterus and to reduce total perioperative blood loss. Arterial balloon occlusion can be applied at different levels, varying from distal placement in the common iliac arteries, internal iliac arteries, or uterine arteries to proximal placement in the abdominal aorta, below the renal arteries. Prophylactic embolization is commonly performed at the distal level, in the internal iliac or uterine arteries. It is postulated that proximal balloon occlusion might be more effective in reducing blood loss because its occlusive effect is countered to a lesser extent by collateral circulation compared with distal occlusion or embolization.^5,12,13^ Reported studies on these endovascular approaches are relatively small in terms of sample size, challenging interpretation of outcomes. In addition, adverse effects associated with these interventions may be severe and include vessel rupture and thromboembolism.^14,15^ Systematically reviewing these study results is needed to support clinical decision making.

The aim of this systematic review and network meta-analysis was to assess whether prophylactic placement of balloon catheters or sheaths before planned cesarean delivery in women with placenta accreta spectrum disorder is associated with reduced perioperative blood loss compared with no prophylactic radiologic intervention.

## SOURCES

This study adhered to the PRISMA (Preferred Reporting Items for Systematic Reviews and Meta-analyses) and MOOSE (Meta-analyses of Observational Studies) reporting guidelines.^16,17^ Before data extraction, this study was registered in PROSPERO (CRD42022320922). The study protocol was approved by the local scientific committee of the Department of Clinical Epidemiology of the Leiden University Medical Center (proposal A167). Together with a certified medical librarian (P.G.), we developed a literature search strategy using key concepts from the research question: “placenta accreta”, “balloon catheter”, “embolization”, and “blood loss” (Appendix 1, available online at http://links.lww.com/AOG/D737). The librarian conducted a comprehensive electronic literature search on January 3, 2023, in the following databases: PubMed, Embase, Cochrane Library, and Web of Science. We also checked ClinicalTrials.gov retrospectively.

Two independent reviewers (L.R.B. and K.S.) screened titles and abstracts to identify potentially eligible records, and full texts of the selected publications were reviewed to assess eligibility. Disagreements were solved through discussion (L.R.B. and K.S.) and, if necessary, by the senior researcher (D.D.C.A.H). Reasons for exclusion were recorded.

## STUDY SELECTION

Studies were eligible if they 1) included pregnant women at risk (discussed later) of placenta accreta spectrum disorder or with postpartum confirmed placenta accreta spectrum disorder diagnosis who underwent a planned cesarean delivery and 2) compared maternal outcomes between women who received prophylactic radiologic interventions and women who did not receive any of these interventions. We considered the following women at risk of placenta accreta spectrum disorder: 1) women with one or more previous cesarean deliveries and a current pregnancy with anterior low-lying placenta or placenta previa and 2) women with ultrasonographic signs of placenta accreta spectrum disorder. *Confirmed placenta accreta spectrum disorder diagnosis* was defined as clinical confirmation of the diagnosis during cesarean delivery by the managing obstetrician–gynecologist or confirmation after histopathologic analysis. We excluded studies in women who underwent emergency cesarean delivery because the emergency setting was hypothesized not to allow time for a prophylactic intervention.

**Fig. 1.**
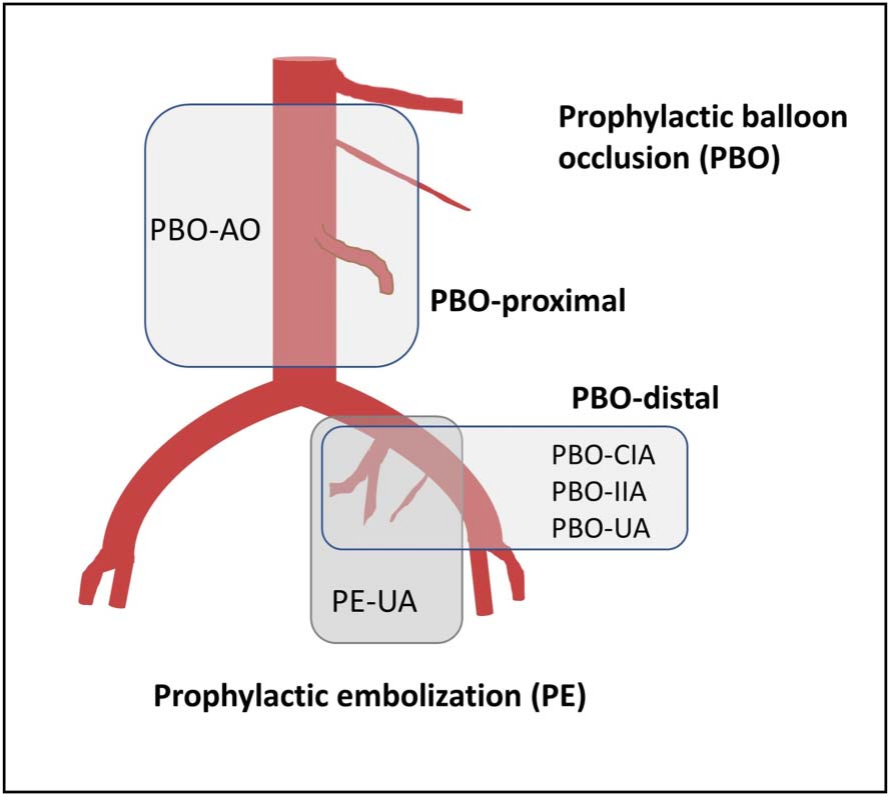
Classification of prophylactic radiologic interventions. AO, abdominal aorta; CIA, common iliac arteries; IIA, internal iliac arteries; UA, uterine arteries.

*Prophylactic radiologic interventions* were defined as follows: 1) preoperative placement of balloon catheters into common iliac arteries, internal iliac arteries, uterine arteries, or abdominal aorta classified into two groups, prophylactic balloon occlusion distally (common iliac arteries, internal iliac arteries, uterine arteries) and proximally (abdominal aorta); and 2) preemptive vascular access with sheaths in the common femoral artery for prophylactic embolization of the uterine arteries (Fig. 1). Randomized controlled trials and observational studies published before January 2023 were eligible. We did not use language restrictions.

*Bonsen. Prophylactic Radiologic Interventions in Women With PAS. Obstet Gynecol 2024*.

The primary outcome was the volume of perioperative blood loss in milliliters. Secondary outcomes were the number of red blood cell (RBC) units transfused within 24 hours after childbirth, maternal mortality, adverse events related to the studied radiologic intervention, and surgical complications. We used the Crown initiative core outcome set for treatment of postpartum hemorrhage and the World Health Organization Maternal Near Miss approach to define outcomes of interest.^18,19^ We aimed to collect data on all outcomes as suggested, including shock, transfer to a higher level of care, use of additional hemostatic interventions, coagulopathy, presence of organ dysfunction, and patient-reported outcomes. As opposed to the core outcome set for treatment of postpartum hemorrhage, we decided not to report hysterectomy as a secondary outcome because we expected most women to have undergone planned cesarean hysterectomy, although the hysterectomy itself might also have been unplanned and performed to stop the bleeding.

Two reviewers (L.R.B. and K.S.) independently extracted the following data: characteristics of included women (age, parity, number of previous cesarean deliveries, type of placenta accreta spectrum disorder), details pertaining to the intervention (number of women with intervention, localization of balloon catheter, procedure of checking position of catheter after transfer to operating theater, inflation of balloon catheter [yes or no], fluoroscopy-guided inflation [yes or no], type and size of balloon catheter, angiography [yes or no], embolization [yes or no], material used for embolization), characteristics of included studies (name of first author, year of publication, study design, single center or multicenter, country or countries of study, total sample size, type and source of financial support, publication status from trial reports, blinding), and outcomes (volume of blood loss, methods of measuring volume of blood loss, mortality and morbidity, use of additional interventions, use of resources, patient-reported outcomes, adverse effects).

For the two continuous outcomes, blood loss (milliliters) and RBC transfusion (units), we expressed effect sizes as mean differences and 95% CIs. If the mean was not reported, we used the median; if the SD was not reported, we derived the SD using the method developed by Wan et al.^20^ According to this method, we used interquartile ranges and sample size to calculate the SD; if interquartile range was not available, we used range. In the included studies, RBC transfusion was reported either as number of units or as a volume. We present this outcome as RBC units. If the authors did not specify the volume of an unit, we assumed it to be 300 mL.

In our primary analysis, the study effect sizes were synthesized with a random-effects frequentist network meta-analysis.^21^ Within this network, we made pairwise comparisons between the three interventions (prophylactic balloon occlusion–distal, prophylactic balloon occlusion–proximal, and prophylactic embolization of the uterine arteries) and the control group. The control group was set as reference. Results are presented in forest plots with corresponding 95% CIs.

Furthermore, mean differences were pooled over studies with the use of pairwise random-effects meta-analyses in which the different interventions were compared with the control situation. With these separate analyses, the results per study are presented in a forest plot combined with the traffic light plot of the risk-of-bias assessment.

Heterogeneity was assessed by the between-trial-variance (τ) with prediction intervals, which presents the expected range of effects in individual studies.^22^ In addition, we report the generalized *I*^2^ statistic for network meta-analysis and pairwise random-effects meta-analysis, which describes the proportion of variability between trials not attributable to chance. Consistency was explored by comparing direct and indirect estimates of the parameters. We performed a sensitivity analysis excluding studies considered at critical and serious risk of bias using the Cochrane Risk of Bias in Non-randomized Studies of Interventions tool.

Two predefined subgroup analyses for the primary outcome blood loss were 1) placenta percreta compared with other types of placenta accreta spectrum disorder, either clinically or histologically identified; and 2) studies that included only women with confirmed placenta accreta spectrum disorder. Meta-analyses was performed with the meta and netmeta packages in R.^21^

Two reviewers (L.R.B. and D.H.) assessed the risk of bias of individual studies independently using the Cochrane Risk of Bias in Non-randomized Studies of Interventions tool.^23^ Disagreements were resolved by discussion with a third reviewer (J.G.v.d.B.). In observational studies addressing our research question, we expected confounding by indication to be an important source of bias. We considered the expected disease severity, placenta percreta (yes or no), to be the most important confounding variable. Results of the risk-of-bias assessment are shown in a traffic light plot created with the robvis tool.^24^ Details on the risk-of-bias assessment are presented in Appendix 2, available online at http://links.lww.com/AOG/D737. We generated a funnel plot to investigate small sample bias for our primary outcome. We also report the Egger test.

## RESULTS

**Fig. 2.**
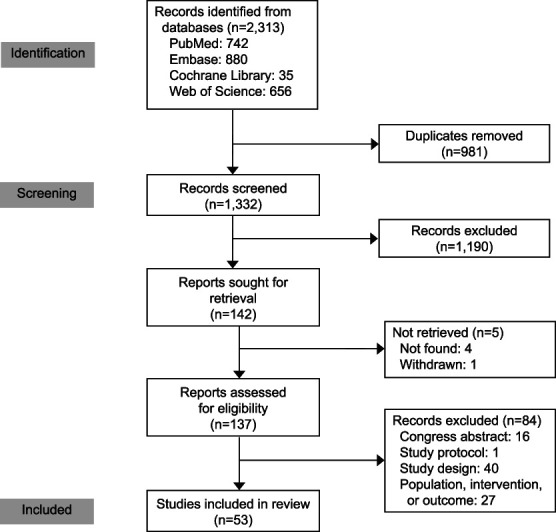
PRISMA (Preferred Reporting Items for Systematic Reviews and Meta-analysis) flow diagram.

The literature search yielded 2,312 citations, of which 1,332 were unique. Figure 2 shows the study selection. In total, 53 studies (6,091 women) were included: two randomized controlled trials (RCTs; 127 women) and 51 observational studies (5,964 women). We analyzed 50 studies numerically; two studies^25,26^ were excluded because of missing SEs for the primary and secondary outcomes, and one study^27^ was excluded because the reported outcome was postpartum hemorrhage of more than 1,000 mL without information on estimated blood loss in milliliters. Study characteristics are shown in Table 1, and details on ultrasonographic criteria used for placenta accreta spectrum disorder diagnosis are shown in Appendix 3, available online at http://links.lww.com/AOG/D737.

*Bonsen. Prophylactic Radiologic Interventions in Women With PAS. Obstet Gynecol 2024*.

**Table 1.**
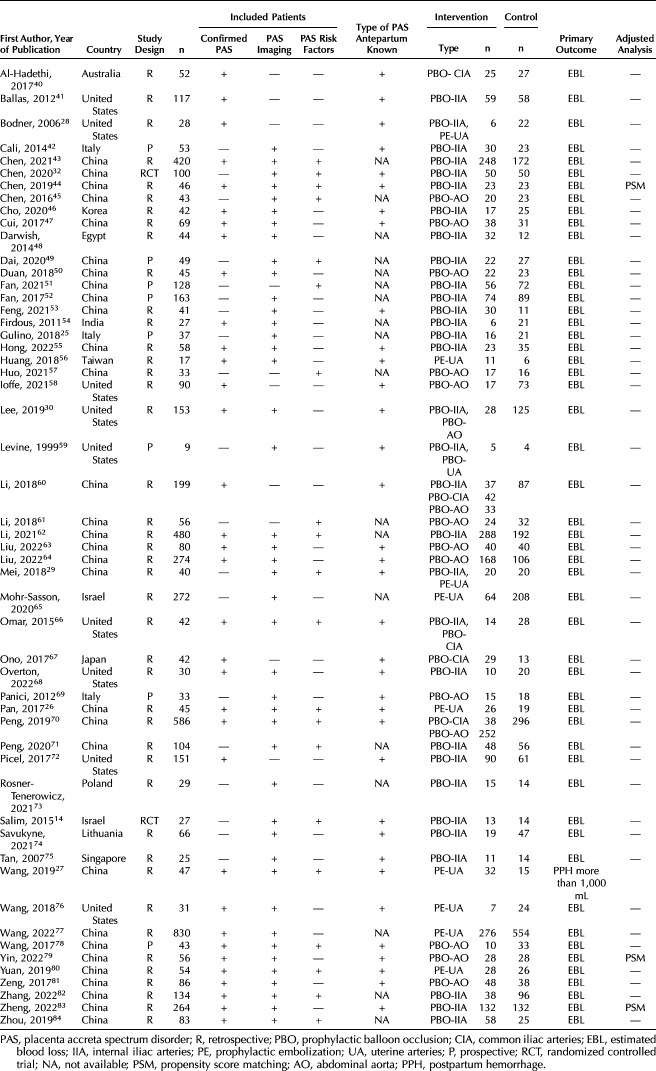
Characteristics of the Included Studies

**Fig. 3.**
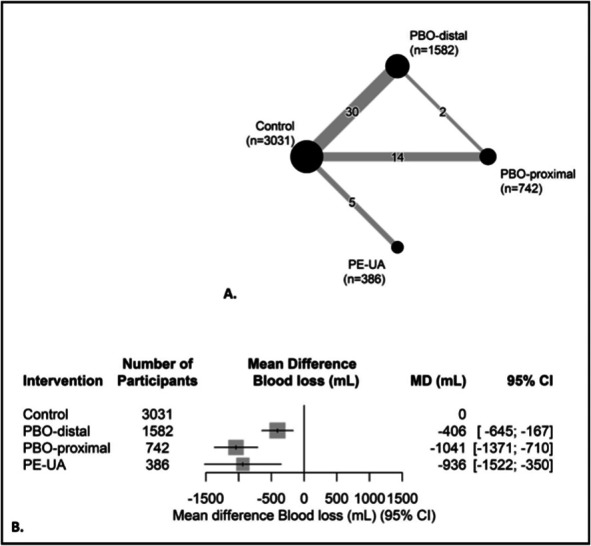
Network meta-analysis primary outcome: blood loss. **A**. Network graph. *Black dots* represent the type of intervention with number of women (n); *gray lines* represent the pairwise comparisons with the number of studies that evaluated a specific comparison (ie, a single study with more than one comparison can be shown multiple times in the figure). **B**. Forest plot: results of the random-effects model. Number of included studies, n=47; heterogeneity: *I*^2^=96%, τ=571 mL. PBO, prophylactic balloon occlusion; PE-UA, prophylactic embolization of the uterine arteries; MD, mean difference.

The network graph (Fig. 3) shows the pairwise comparisons within the network meta-analysis. Thirty studies compared postpartum blood loss in women with prophylactic balloon occlusion–distal (n=1,582) with a control group without prophylactic radiologic intervention. Fourteen studies compared prophylactic balloon occlusion–proximal with a control group, and five studies evaluated prophylactic embolization of the uterine arteries. Three studies were excluded from the main analysis because the data could not be separated per intervention: two studies of prophylactic balloon occlusion–distal or prophylactic embolization of the uterine arteries^28,29^ and one study of prophylactic balloon occlusion–distal or –proximal.^30^ Therefore, the sample sizes were very small. A sensitivity analysis including these three studies is presented in Appendix 4, available online at http://links.lww.com/AOG/D737. Details of the prophylactic radiologic interventions are shown in Appendix 5, available online at http://links.lww.com/AOG/D737.

*Bonsen. Prophylactic Radiologic Interventions in Women With PAS. Obstet Gynecol 2024*.

Figure 3 shows the results for the primary analysis (network meta-analysis).Women with prophylactic radiologic interventions had on average a lower volume of perioperative blood loss compared with the control group. The mean differences were −406 mL (95% CI, −645 to −167) for prophylactic balloon occlusion–distal, −1,041 mL (95% CI, −1,371 to −710) for prophylactic balloon occlusion–proximal, and −936 mL (95% CI, −1,522 to −350) for prophylactic embolization of the uterine arteries. Heterogeneity *I*^2^ was 96%, and τ was 571 mL.

**Fig. 4.**
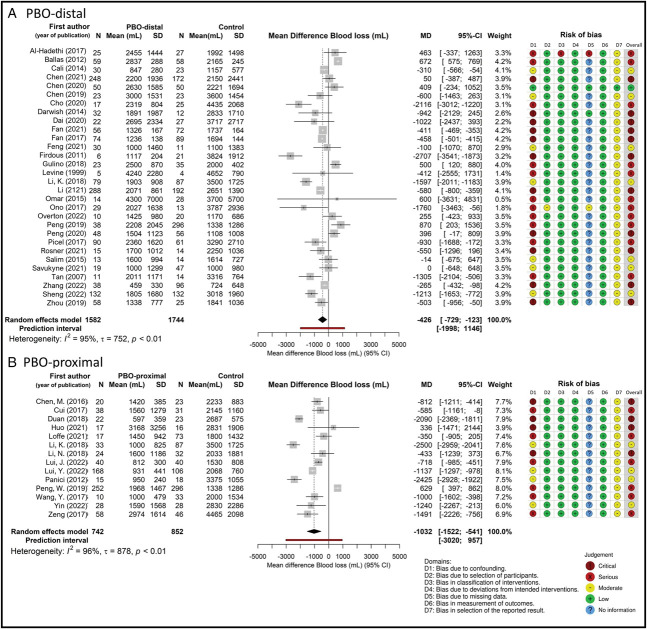
Forest plot of meta-analysis per intervention for primary outcome blood loss and risk-of-bias assessment. Prophylactic balloon occlusion (PBO)–distal (**A**) and PBO-proximal (**B**). *Results of intervention prophylactic embolization-uterine arteries are presented in Appendix 6, available online at http://links.lww.com/AOG/D737. MD, mean difference.

Results of the pairwise analysis per prophylactic radiologic intervention, along with the risk-of-bias judgment per study, are presented in Figure 4. In studies that compared outcomes of women with prophylactic balloon occlusion–distal with outcomes from a control group, the mean difference in blood loss was −426 mL (95% CI, −729 to −123, *I*^2^=95%, τ=752 mL) (Fig. 4A). In studies comparing prophylactic balloon occlusion–proximal with a control group, the mean difference was −1,032 mL (95% CI, −1,522 to −541, *I*^2^=96%, τ=878 mL) (Fig. 4B). Finally, in studies that compared prophylactic embolization of the uterine arteries with a control group, the mean difference was −1,216 mL (95% CI, −2,637 to 205, *I*^2^=98%, τ=1,568 mL) (Appendix 6, available online at http://links.lww.com/AOG/D737).

*Bonsen. Prophylactic Radiologic Interventions in Women With PAS. Obstet Gynecol 2024*.

Within the network meta-analysis, there were three comparisons that used both direct and indirect comparisons. The difference between the direct and indirect estimate was not statistically significant for either of the comparisons.

The results of our subgroup analysis on type of placenta accreta spectrum disorder are presented in Appendix 7, available online at http://links.lww.com/AOG/D737. Eight studies reported placenta percreta. In women with placenta percreta, the mean difference in blood loss for women with prophylactic balloon occlusion–distal (n=204) compared with control (n=250) was −792 mL (95% CI, −1,288 to −296, *I*^2^=48%, τ=335 mL).

Almost two-thirds of the studies in this meta-analysis (n=31) had selected women with placenta accreta spectrum disorder that was confirmed postpartum. In this subgroup, the mean difference in blood loss comparing prophylactic radiologic interventions with a control group was −494 mL (95% CI, −929 to −59) in women with prophylactic balloon occlusion–distal (n=1,180), −1,044 mL (95% CI, −1,581 to −507) in women with prophylactic balloon occlusion–proximal (n=666), and −1,715 mL (95% CI, −2,646 to −784) in women with prophylactic embolization of the uterine arteries (n=322) (Appendix 8, available online at http://links.lww.com/AOG/D737).

Thirty-five studies reported data on RBC transfusion. According to a network meta-analysis, women with prophylactic radiologic interventions received, on average, a lower number of RBC units transfused: mean difference of −1.13 (95% CI, −2.27 to 0.02) for prophylactic balloon occlusion–distal, −1.90 (95% CI, −3.55 to 0.25) for prophylactic balloon occlusion–proximal, and −1.86 (95% CI, −4.52 to 0.80) for prophylactic embolization of the uterine arteries (Appendix 9, available online at http://links.lww.com/AOG/D737).

The included studies presented limited information about indications for hysterectomy. We present the reported information on the number of hysterectomies per intervention in Appendix 10, available online at http://links.lww.com/AOG/D737. In addition, we documented the number of women in studies who had only planned hysterectomy. The majority of the included studies did not report any information about our predefined secondary outcomes, including shock, transfer to a higher level of care, coagulopathy, organ dysfunction, and patient-reported outcomes. Therefore, we did not report results on these secondary outcomes.

Adverse events reported in the included studies are summarized in Appendix 11, available online at http://links.lww.com/AOG/D737. Thirty-nine studies reported data on adverse events related to prophylactic radiologic intervention, and 36 reported data on surgical complications.

In the prophylactic balloon occlusion–distal group (n=953), 21 adverse events (≈2%) related to the prophylactic radiologic intervention were reported, with thrombus formation in nine patients. Appendix 11 (http://links.lww.com/AOG/D737) presents the percentage of the total number of adverse events divided by number of women. This might be an overestimation because multiple adverse events can occur in one patient. We classified complications according to the Cardiovascular and Interventional Radiological Society of Europe classification system, acknowledging that not all adverse events were categorized because of the unavailability of data.^31^

Sixteen adverse events (≈2%) occurred in the prophylactic balloon occlusion–proximal group, with thrombus formation in 14 patients. In the prophylactic embolization of the uterine arteries group, 42 adverse events (≈45.2%) related to the prophylactic intervention occurred, and 32 of these were lumbosacral pain. Appendix 11 (http://links.lww.com/AOG/D737) also presents surgical complications per intervention. One maternal death attributed to diffuse intravascular coagulation was reported. Three women with a cardiac arrest were reported across different studies, all in control groups and all with successful resuscitation.

According to our risk-of-bias judgments, 18 studies were rated as critical, 23 studies as serious, 11 studies as moderate, and one study as low (Fig. 4 shows prophylactic balloon occlusion–distal and –proximal, and Appendix 6, http://links.lww.com/AOG/D737, shows prophylactic embolization of the uterine arteries). Publication bias was unlikely according to both visual inspection of the funnel plot and the Egger test (Appendix 12, available online at http://links.lww.com/AOG/D737). The sensitivity analysis in studies with low to moderate risk of bias (n=11) showed that the mean difference in blood loss in women with prophylactic balloon occlusion–distal (n=376) was −447 mL (95% CI, −920 to 27), comparable with the primary analysis, and in women with prophylactic balloon occlusion–proximal (n=244) was −1,708 mL (95% CI, −2,351 to −1,065) (Appendix 13, available online at http://links.lww.com/AOG/D737).

## DISCUSSION

The present systematic review and network meta-analysis shows that prophylactic radiologic interventions placed before a planned cesarean delivery in women with placenta accreta spectrum disorder were associated with reduced perioperative blood loss and less RBC transfusion. This association was observed for prophylactic balloon occlusion–distal and –proximal and for prophylactic embolization and was most pronounced among women with confirmed placenta percreta. Considerable heterogeneity across studies precludes the generalizability of the overall estimated effect to different severities of placenta accreta spectrum disorder.

Previous studies have shown inconsistent findings regarding the efficacy of prophylactic radiologic interventions to reduce peripartum blood loss in women at risk of placenta accreta spectrum disorder. Notably, two single-center RCTs have investigated the use of prophylactic distal balloon catheters in women with placenta accreta spectrum disorder. Neither study observed a reduction in blood loss attributable to the intervention.^14,32^ An explanation for this might be the small sample size and the low number of placenta percreta cases in the first published RCT (2015).^14^ In the most recent RCT (2020), the absence of an effect may have been the result of unsuccessful randomization, leading to a disbalance in the number of placenta percreta.^32^

Observational studies of different types of prophylactic radiologic interventions have been assessed as an intrapartum management strategy to reduce postpartum hemorrhage in women with placenta accreta spectrum disorder. These interventions include prophylactic balloon occlusion (distal or proximal) and prophylactic embolization (distal). In the present network meta-analysis, we merged the results of all studies with pairwise comparisons of one or more of the interventions and a control group. We did not predefine the aim to determine treatment ranks; thus, we do not present data on this aspect.

A strength of our study compared with the prior literature was our thorough assessment of the risk of bias in individual studies.^12,33–35^ We used the Cochrane Risk of Bias in Non-randomized Studies of Interventions tool, which covers seven bias domains extensively.^23^ As illustrated in Figure 4, the most critical domain in our study was domain 1: bias attributable to confounding. We considered *disease severity*, defined as placenta percreta (yes or no), the most crucial confounding variable. We assumed that women with a more severe type of placenta accreta spectrum disorder were more likely to receive a prophylactic radiologic intervention and that these women were more likely to have more severe postpartum hemorrhage. This bias could lead to an underestimation of the effect. Potential confounding by disease severity could not be an explanation for the protective association of the prophylactic radiologic intervention that we found in the present meta-analysis. Yet, other confounding variables such as hospital-specific characteristics or the fact that some studies used a historical cohort as a comparison could explain this result. In both situations, treatment variation could cause confounding. Our sensitivity analysis in which we included only studies with low or moderate risk of bias (n=11) showed stable results for prophylactic balloon occlusion–distal and prophylactic balloon occlusion–proximal. All studies on prophylactic embolization of the uterine arteries (n=5) were judged as having serious or critical risk of bias and were therefore excluded.

An important result was the high level of heterogeneity between the included studies. This is shown by both the between-trial variance (τ^2^) with prediction interval and the *I*^2^ statistic, which is the percentage of total variation across studies that is the result of heterogeneity rather than chance. The most likely explanation for the heterogeneity is that placenta accreta spectrum disorder is characterized by a wide range of severities, from placenta accreta to the most severe type, placenta percreta. This is corroborated in our subgroup analysis focusing on placenta percreta in which the heterogeneity was markedly lower (*I*^2^=48%). Unfortunately, only a very limited number of the included studies could be included in this subgroup analysis (n=8). Because placenta accreta spectrum disorder is such a heterogeneous condition, one should not aim to generalize the overall estimated effect to different severities of placenta accreta spectrum disorder. A second possible explanation for the heterogeneity resides in differences in treatments across studies and across countries. We aimed to compare women with placenta accreta spectrum disorder who received prophylactic radiologic interventions with women who did not receive such interventions. To make this comparison, ideally one should assume that all women received standard care for postpartum hemorrhage according to similar guidelines. Different surgical approaches, including planned hysterectomy or uterine-preserving surgery, could also explain the heterogeneity.^36–38^ Unfortunately, we were not able to study hysterectomy as an outcome because data on the indication were not available in the majority of the studies. Furthermore, we were unable to collect data on most of our predefined secondary outcome measures because the majority of the included studies did not report any information on these outcomes. The use of blood loss as a primary outcome has its known limitations; there exists a level of inaccuracy in estimating the volume of postpartum blood loss.^39^ In addition, there was variation in the methodology of blood loss estimation between studies. We think this might have affected the results within studies, but we believe it is unlikely that differences in methodology have a significant effect on our overall results.

Data on adverse events related to the radiologic intervention were not reported by all studies. Consequently, we cannot make definitive statements about safety. In the studies that did report adverse events of the radiologic intervention, about 2% of women experienced such events in both the prophylactic balloon occlusion–proximal and prophylactic balloon occlusion–distal groups. Because of the large variability in the reporting of adverse events across studies, caution is warranted in comparisons of the percentage of adverse events in the prophylactic embolization of the uterine arteries group (12%).

There was substantial variation in inclusion criteria between studies. Thirty-one studies included women with a confirmed postpartum diagnosis, constituting a subgroup within the population of interest for this systematic review. This distinction is crucial because the decision to use a prophylactic radiologic intervention is always made antepartum, potentially leading to the inclusion of women who did not get a postpartum placenta accreta spectrum disorder diagnosis. Our subgroup analysis of women with confirmed placenta accreta spectrum disorder revealed results similar to those of our main analysis.

Our main analysis reveals differences in outcomes among the three interventions, with proximal balloon occlusion demonstrating the strongest effect. Our results show a blood loss reduction of 406 mL by distal prophylactic balloon occlusion. An explanation for the differences between the results of prophylactic balloon occlusion–distal and prophylactic balloon occlusion–proximal could be that implementing occlusion at a distal level may be less effective because of bleeding from the collateral circulation.^2,5^ In this study, we included only five studies on prophylactic embolization, all of which had severe or critical risk of bias. This, in combination with the wide CI as a result of the small sample size, limited our ability to interpret the results of this intervention.

In sum, we believe that this critical overview of the available evidence provides valuable insights for clinical decision making. Our study highlights that, if we were to be certain of the diagnosis of placenta accreta spectrum disorder antepartum, prophylactic radiologic intervention could help reduce peripartum blood loss. However, the current limitation lies in the suboptimal accuracy of antepartum diagnosis, leading to potential overtreatment of women.

In conclusion, although the predominance of observational studies in the included literature warrants caution in the interpretation of the findings of this meta-analysis, our findings suggest that prophylactic placement of balloon catheters or sheaths before a planned cesarean delivery in women with placenta accreta spectrum disorder may, in some cases, reduce perioperative blood loss. Further study is required to quantify the efficacy according to various severities of placenta accreta spectrum disorder and the associated safety of these radiologic interventions.
